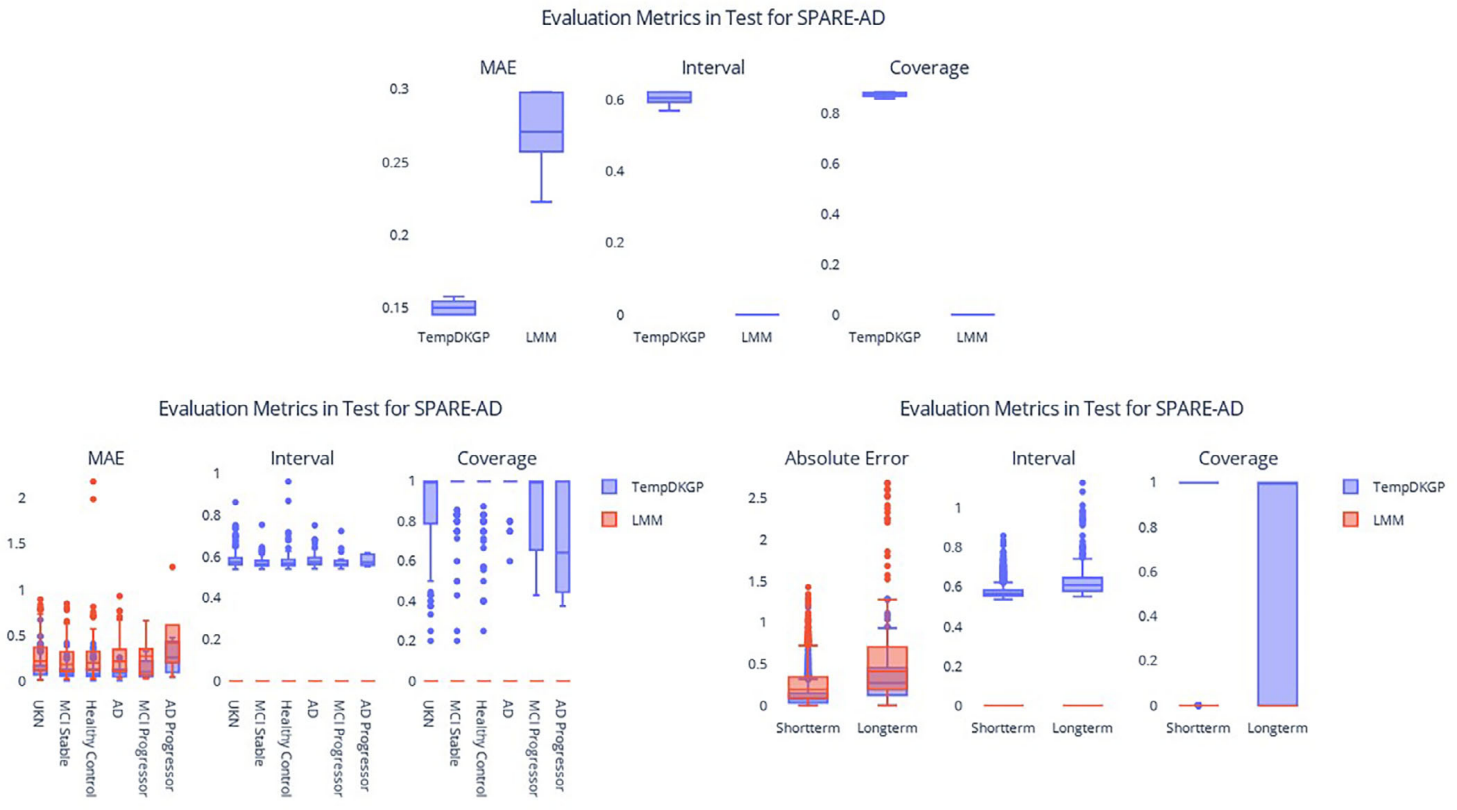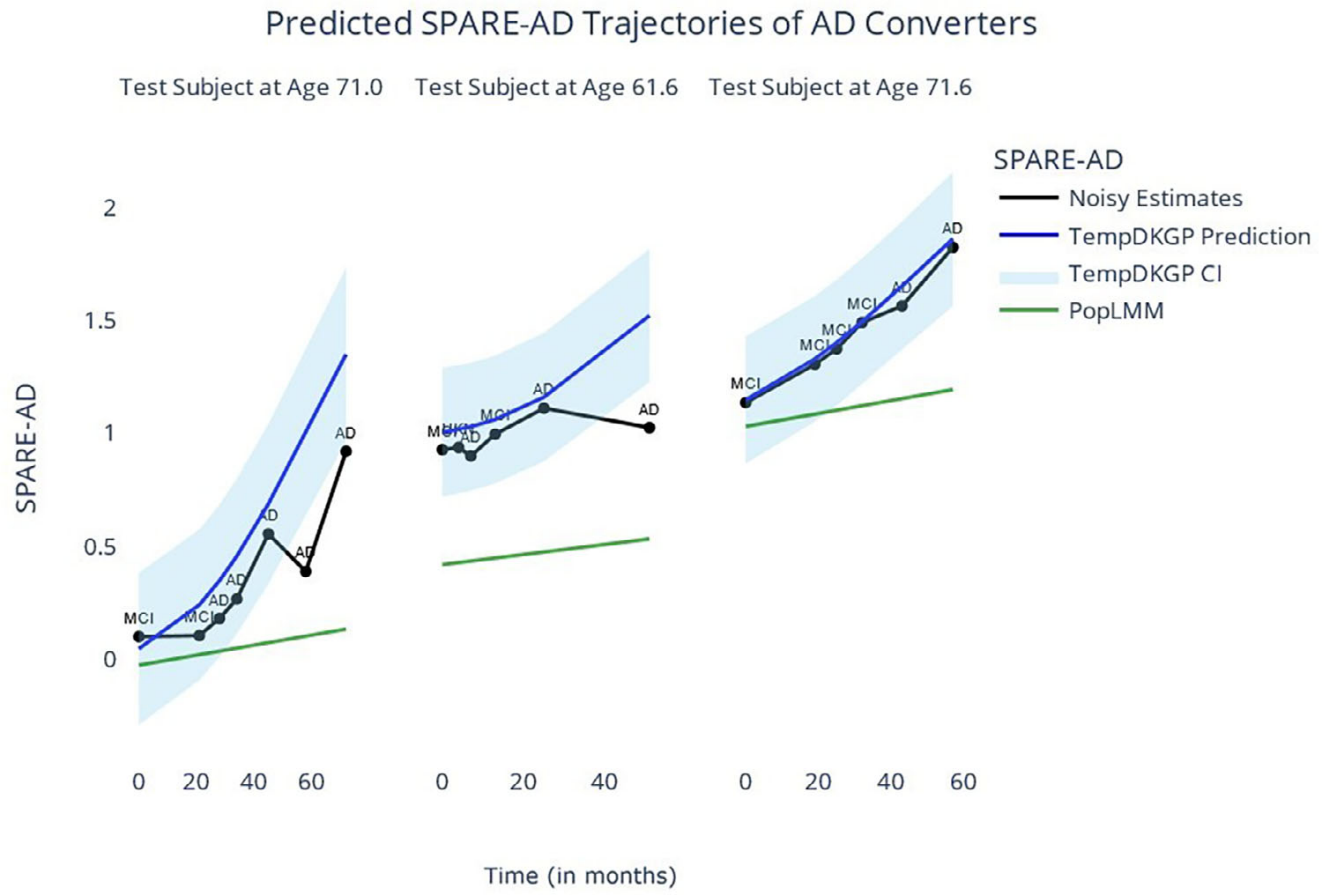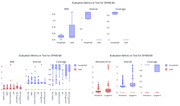# Predicting future Biomarker changes in Alzheimer's Disease, Brain Aging and Comorbidities

**DOI:** 10.1002/alz.089473

**Published:** 2025-01-09

**Authors:** Vasiliki Tassopoulou

**Affiliations:** ^1^ Artificial Intelligence in Biomedical Imaging Laboratory (AIBIL), Center for and Data Science for Integrated Diagnostics (AI2D), Perelman School of Medicine, University of Pennsylvania, Philadelphia, PA USA

## Abstract

**Background:**

The Spatial Pattern of Abnormality for REcognition of Alzheimer's Disease (SPARE‐AD) index ∖citep{davatzikos2009longitudinal} is one such marker that robustly discriminates between early brain changes observed in cognitively normal aging (CN), mild cognitive impairment (MCI), and Alzheimer's Disease (AD) phenotypes. The adoption of such markers to the clinical setting combined with the ability to forecast their future trajectories would be of great value during clinical assessment, and could improve clinical trial design through targeted risk stratification.

**Method:**

Subjects scanned using the same scanner with more than four longitudinal MRI acquisitions from the Alzheimer's Disease Neuroimaging Initiative (ADNI) and Baltimore Longitudinal Study of Aging (BLSA) study cohorts were used for method development. T1 structural MRIs were preprocessed and segmented into 145 gray (GM) and white matter (WM) regions of interest (ROIs) using previously described methods. The output of the model is the temporal function of the biomarker score, which in this work is the SPARE‐AD and the SPARE‐BA, seperately. The core of our methodological advancement revolves around the concept that the progression of each biomarker can be represented by a temporal function, f, which is approximated with a Gaussian Process (GP). The learned temporal function is a function of the baseline acquisition of the subject, which means that on inference time, we need only the first scan of the subject so as to get the temporal function of the biomarker

**Result:**

The Temporal Deep Kernel GP (TempDKGP) model achieves higher predictive performance on both SPARE‐AD and SPARE‐BA model. Our model had lower MAE averaged across all participants per K‐fold when compared to ithe LMM. MAEs were also lower when the cohort was subgrouped by their longitudinal cognitive status (i.e., stable vs progression), and by short‐term (less than 5 years ahead of baseline) or long‐term predictions (more than 5 years ahead of baseline). Longitudinal trajectories predicted using baseline features by TempDKGP more closely followed the ground truth trajectories than the LMM as illustrated.

**Conclusion:**

The TempDKGP model is a predictive model that extrapolates biomarker trajectories along with uncertainty intervals.